# Spheroid assembly of mesenchymal stem cells enhances secretome-mediated corneal reinnervation and epithelial repair in a mouse model of experimental dry eye

**DOI:** 10.1177/20417314251363300

**Published:** 2025-08-19

**Authors:** Shao-Wen Liu, Meng-Yu Tsai, Yang-Chun Shen, Yi-Jen Hsueh, Han Chiu, Li-Wen Hsu, Hung-Chi Chen, Chieh-Cheng Huang

**Affiliations:** 1 Institute of Biomedical Engineering, National Tsing Hua University, Hsinchu, Taiwan; 2 Department of Ophthalmology, Linkou Chang Gung Memorial Hospital, Taoyuan, Taiwan; 3 Center for Tissue Engineering, Linkou Chang Gung Memorial Hospital, Taoyuan, Taiwan; 4 International PhD Program in Innovative Technology of Biomedical Engineering and Medical Devices, Ming Chi University of Technology, New Taipei City, Taiwan; 5 Bioresource Collection and Research Center, Food Industry Research and Development Institute, Hsinchu, Taiwan; 6 Department of Medicine, Chang Gung University, Taoyuan, Taiwan

**Keywords:** mesenchymal stem cell, stem cell secretome, cell spheroid, corneal nerve, ocular surface regeneration

## Abstract

Dry eye disease is a complex ocular surface disorder with multifactorial pathophysiology, including corneal epithelial damage, chronic inflammation, and corneal nerve dysfunction. Among these, impaired corneal innervation plays a particularly critical role, as it disrupts neurotrophic support and tear reflexes, perpetuating disease progression, and delaying healing. However, conventional treatments often provide only temporary symptom relief without addressing underlying tissue damage or promoting nerve regeneration. This shortcoming highlights the need for therapies that not only suppress inflammation but also restore corneal innervation. In this study, we evaluated the therapeutic potential of mesenchymal stem cell (MSC) spheroid-derived secretome—a cell-free solution rich in regenerative and anti-inflammatory factors—in a preclinical mouse model of dry eye disease. Compared with untreated controls, eyes treated with the MSC spheroid secretome presented faster corneal epithelial regeneration, improved corneal nerve reinnervation, and reduced inflammatory cell infiltration. These findings demonstrate that the MSC spheroid-derived secretome can simultaneously target multiple pathological features of dry eye to promote recovery of ocular surface integrity, underscoring its potential as a clinically relevant, cell-free regenerative therapy for dry eye and other ocular surface disorders.

## Introduction

The cornea functions as the primary refractive surface of the eye by bending incoming light to focus it on the retina, enabling clear vision.^1^ It is composed of three principal layers, the epithelium, stroma, and endothelium, which together maintain the structural integrity and optical transparency of the tissue.^1^ In addition to its optical role, the cornea is densely innervated by sensory nerve fibers originating from the ophthalmic branch of the trigeminal (TG) nerve.^2^ These corneal nerves fulfill both sensory and trophic functions, contributing to epithelial cell proliferation, wound healing, and tear secretion.^3–5^ This neuroanatomical interaction is critical for preserving corneal homeostasis, and the disruption of this interaction can predispose the ocular surface to a range of disorders, most notably dry eye disease.^1,6^

Dry eye disease is a complex, multifactorial disorder characterized by tear film instability, hyperosmolarity, chronic inflammation, oxidative stress accumulation, and neurosensory abnormalities.^1,7–9^ Affecting nearly 30% of the global population, dry eye disease imposes a substantial socioeconomic burden.^1,3,6,9^ Patients with dry eye disease often present with ocular discomfort, burning sensations, fluctuating vision, and an increased risk of corneal injury.^1,3,7,10^ The pathophysiology of dry eye disease involves a self-sustaining vicious cycle in which tear film instability induces hyperosmolar stress, triggering corneal and conjunctival epithelial cells to release proinflammatory cytokines and reactive oxygen species (ROS)^9–12^; this response promotes leukocyte infiltration, further amplifying local inflammation and oxidative stress, ultimately leading to epithelial damage and corneal nerve injury.^9–13^ As a result, neurotrophic support is compromised, and tear production via the lacrimal reflex is reduced, further destabilizing the tear film.^14^ Persistent oxidative stress also contributes to progressive corneal nerve degeneration, increasing the vulnerability of the ocular surface and delaying wound healing.^9,15,16^ If left untreated, this cascade of inflammation, oxidative damage, and nerve dysfunction can progress to advanced complications such as neurotrophic keratopathy, corneal ulceration, and vision impairment.^11,17,18^

Current treatments for dry eye disease offer only limited symptomatic relief and do little to halt the progression of the underlying disease.^19^ Artificial tears provide transient lubrication but do not address the inflammatory and neurotrophic mechanisms at the core of the disease.^1,19,20^ Anti-inflammatory agents such as cyclosporine A and lifitegrast can dampen ocular surface inflammation, but their efficacy is often modest, with a delayed onset of action.^13,20,21^ Many patients report only partial symptom improvement after months of use, and real-world data show that more than 60% of patients discontinue therapy within the first year because of insufficient relief or adverse effects.^22^ These poor adherence rates underscore the limitations of current pharmaceuticals, which are often poorly tolerated and fail to produce meaningful, sustained benefits.^22^

More critically, none of the approved therapies repair the structural damage or nerve loss that underlies persistent symptoms.^5,23^ Anti-inflammatory drops may suppress inflammation but do not actively promote corneal reinnervation^24^—leaving the root causes of dry eye disease unaddressed. Corneal nerves regenerate slowly, often taking months to years to recover even partially, during which patients remain prone to recurrent injury and chronic discomfort.^4,25,26^ These therapeutic gaps highlight the urgent need for regenerative strategies that both suppress inflammation and restore tissue and nerve integrity. Given the global prevalence, chronic morbidity, and rising economic burden of dry eye disease, the development of more effective, disease-modifying treatments is both timely and essential.^1,27^

Mesenchymal stem cells (MSCs) have emerged as compelling candidates for regenerative therapy because of their capacity to secrete a broad spectrum of bioactive molecules, including growth factors, cytokines, and extracellular vesicles.^28^ By acting primarily through paracrine signaling, MSCs exert immunomodulatory, neuroprotective, and tissue-regenerative effects, making them particularly well-suited for the treatment of inflammatory and degenerative conditions.^28,29^ In ocular surface diseases, MSCs have been shown to suppress inflammation, accelerate epithelial wound healing, and promote corneal nerve regeneration.^30–34^ However, the direct application of MSCs to the ocular surface presents several challenges, including poor cell retention, limited survival in the inflamed microenvironment, and inconsistent therapeutic outcomes.^35–37^ In addition, invasive delivery routes such as intracameral injection can be technically demanding and may lead to patient discomfort or procedural risks.

To address these limitations, MSC-derived biologics—particularly the MSC secretome—have emerged as promising cell-free alternatives.^35–37^ The secretome, which is composed of soluble factors (such as growth factors and cytokines) and extracellular vesicles released by MSCs,^38^ retains the immunomodulatory and regenerative properties of the parent cells.^35–37^ Preclinical studies have shown that the MSC secretome can effectively suppress ocular surface inflammation and promote epithelial repair.^33,39–42^ Compared with cell-based therapies, secretome-based approaches offer distinct advantages, including improved safety, greater stability, and ease of administration.^33,35^ When delivered as eye drops, the MSC-derived secretome offers a noninvasive, patient-friendly platform with adjustable dosing schedules, making it especially suitable for managing chronic conditions such as dry eye disease.^35,43^

However, despite encouraging progress with secretome-based therapies, conventional approaches relying on the secretome derived from two-dimensional (2D) monolayer cultures have shown limited efficacy in restoring corneal structure and homeostasis.^35,39,42,43^ While these cell-free treatments offer benefits, they often fail to address the multifactorial nature of dry eye disease. In particular, one of the most critical limitations of existing studies is their predominant focus on epithelial repair and immunomodulation, with little attention given to corneal nerve regeneration.^35,39,42,43^ This represents a significant therapeutic gap, as corneal innervation plays an essential role in maintaining epithelial health, modulating inflammation, and supporting wound healing.^1,3–6^ The uncertain neuroregenerative capacity of conventional MSC secretome therapies underscores the need for novel strategies—particularly in light of the central role of nerve damage and neurotrophic dysfunction in the pathogenesis and persistence of dry eye disease.

To address these limitations directly and improve therapeutic outcomes, the present study not only evaluated the regenerative potential of MSC-derived secretome in a preclinical dry eye model but also, for the first time, investigated its efficacy in promoting corneal nerve regeneration using the secretome derived from MSC spheroids—a strategy developed to increase their paracrine activity.^44,45^ Unlike traditional 2D monolayer cultures, MSC spheroids are three-dimensional (3D) aggregates that more closely mimic the native tissue microenvironment.^46–48^ In this configuration, MSCs engage in enhanced cell–cell and cell–matrix interactions, leading to increased paracrine secretion.^46,47,49,50^ Multiple studies, including our own^51–54^ and those of others,^46,49,50^ have shown that the spheroid-derived secretome contains significantly higher levels of growth factors, neurotrophic molecules, and anti-inflammatory cytokines than those in the secretome derived from 2D cultures. This enriched paracrine profile—particularly its superior capacity to modulate inflammation and support neural repair—positions the MSC spheroid-derived secretome as a promising candidate for regenerative applications in dry eye disease, where chronic inflammation and nerve injury are central to disease progression.

Although the therapeutic benefits of the MSC spheroid-derived secretome have been demonstrated in various disease models, its potential in the treatment of dry eye remains largely unexplored. We hypothesize that the spheroid-derived secretome not only outperforms the conventional 2D-derived secretome in promoting corneal regeneration but also provides a more robust protective effect against ocular surface inflammation and nerve damage. In this study, we evaluated the therapeutic efficacy of MSC spheroid-derived secretome on corneal epithelial cells, TG neurons, and macrophages using in vitro models, and further assessed its effects in a mouse model of benzalkonium chloride (BAK)-induced dry eye disease. Specifically, we assessed the capacity of the MSC spheroid-derived secretome to stimulate corneal epithelial proliferation, support nerve regeneration, and attenuate ocular surface inflammation. By demonstrating the therapeutic advantages of the MSC spheroid-derived secretome, our findings establish a strong preclinical rationale for its further development as a cell-free regenerative therapy for dry eye disease. Ultimately, this approach may represent a meaningful advance over existing treatments and offer renewed therapeutic potential for patients affected by this chronic and debilitating condition.

## Materials and methods

### MSC spheroid formation and CM collection

Human MSCs derived from the umbilical cord were obtained from the Bioresource Collection and Research Center (Food Industry Research and Development Institute, Hsinchu, Taiwan). The cells were maintained in α-minimum essential medium (MEM; Thermo Fisher Scientific, Waltham, MA, USA) supplemented with 20% fetal bovine serum (FBS; Corning; Corning, NY, USA), 4 ng/mL basic fibroblast growth factor (PeproTech, Rocky Hill, NJ, USA), 30 µg/mL hygromycin B, and 100 µg/mL geneticin (Thermo Fisher Scientific).^55–57^

To facilitate spheroid formation, MSCs were seeded into 96-well plates (20,000 cells per well) that had been coated with a methylcellulose (Sigma-Aldrich, St. Louis, MO, USA) based hydrogel to create a nonadhesive environment. This surface discouraged cell attachment and facilitated spontaneous three-dimensional aggregation.^58–62^ Following a 24-h incubation period under standard culture conditions, the resulting spheroids were visualized using phase-contrast microscopy. Images were captured and subsequently analyzed with ImageJ software to assess spheroid morphology, including size (diameter) and structural uniformity (circularity).

To assess the effects of spheroid assembly on gene expression, total RNA was extracted from MSCs cultured in either 2D monolayers or spheroids using TOOLSmart RNA Extractor (BIOTOOLS, New Taipei City, Taiwan) following the manufacturer’s instructions. cDNA synthesis was performed using a High-Capacity cDNA Reverse Transcription Kit (Thermo Fisher Scientific). Quantitative polymerase chain reaction (qPCR) was conducted using Genious 2X SYBR Green Fast qPCR Mix (ABclonal, Woburn, MA, USA) and a QuantStudio 3 Real-Time PCR System (Thermo Fisher Scientific). Target gene expression was normalized to that of *RPL13A*.^51,54^ The primer sequences are listed in Table S1.

To generate MSC-CM, 3 × 10^5^ MSCs—either as single-cell suspensions (for 2D culture) or in spheroid form (15 spheroids per well)—were seeded into 6-well plates. After cell attachment, the medium was replaced with 2 mL of fresh medium, followed by incubation for 48 h. The resulting CM was collected, centrifuged at 1000 *× g* for 10 min to remove debris, and stored at −80°C for subsequent use.

### Assessment of corneal epithelial cell proliferation and migration

Human corneal epithelial cells (CRL-11515; ATCC, Manassas, VA, USA) were maintained in keratinocyte serum-free medium supplemented with 5 ng/mL epidermal growth factor (EGF), 0.05 mg/mL bovine pituitary extract, and 0.005 mg/mL insulin (Thermo Fisher Scientific).^42,63^ To evaluate the effects of MSC-CM on epithelial proliferation, cells were seeded at a density of 5000 cells per well in 96-well plates. After overnight attachment, the medium was replaced with either CM obtained from conventional 2D MSC cultures or from MSC spheroids. The control wells received unconditioned medium. To model the inflammatory microenvironment of dry eye disease, 100 ng/mL interleukin (IL)-1β (PeproTech) was added.^64,65^ After 24 h of incubation, cell morphology was observed using phase-contrast microscopy, and cell viability was assessed using cell counting kit (CCK)-8 assays (IMT Formosa New Materials, Kaohsiung, Taiwan).^66^ Alternatively, cell proliferation was assessed using the E-Click 5-ethynyl-2’-deoxyuridine (EdU) Cell Proliferation Imaging Assay Kit (Elabscience; Houston, TX, USA) according to the manufacturer’s instructions. After nuclear staining with 4′,6-diamidino-2-phenylindole (DAPI), the cells were imaged under a fluorescence microscope. The percentage of proliferating cells was calculated as follows: EdU^+^ cells (%) = (number of EdU^+^ cells/number of DAPI^+^ cells) × 100%.

To evaluate the impact of MSC-CM on epithelial cell migration, 15,000 corneal epithelial cells were seeded into 48-well plates and cultured to confluence. A scratch was introduced in each well using a sterile P200 pipette tip.^66^ After rinsing with PBS, the cultures were then incubated with CM from either 2D MSCs or MSC spheroids, whereas the control wells received unconditioned medium. Wound closure was monitored using phase-contrast microscopy at 0, 24, and 48 h after scratching. The residual wound area was quantified using ImageJ software and normalized to the area at baseline (0 h) to calculate the wound closure rate.

### TG neuron isolation and neurite outgrowth assay

All animal procedures were conducted in accordance with the 2018 Guidelines for the Care and Use of Laboratory Animals issued by the Council of Agriculture, Executive Yuan, Taiwan, and adhered to the ARVO Statement for the Use of Animals in Ophthalmic and Vision Research. The experimental protocol was approved by the Institutional Animal Care and Utilization Committee of National Tsing Hua University, Hsinchu, Taiwan (Approval No. 10641).

TG neurons were isolated from eight-week-old male C57BL/6 mice (BioLasco, Taipei, Taiwan) euthanized by CO_2_ inhalation. The ophthalmic branches of the TG were dissected, rinsed in ice-cold Dulbecco’s modified Eagle medium (DMEM)/F-12 (Thermo Fisher Scientific), and enzymatically digested at 37 °C for 20 min in a solution containing 5 mg/mL collagenase type II (Worthington Biochemical, Lakewood, NJ, USA), 0.25 mg/mL trypsin (Thermo Fisher Scientific), 0.2 mg/mL DNase I (Sigma-Aldrich), and 1% antibiotic–antimycotic.^67^ Following digestion, the tissues were gently triturated and filtered through a 70-µm nylon cell strainer to obtain a single-cell suspension. The cells were then centrifuged at 1500 rpm for 5 min and resuspended in DMEM/F-12 supplemented with 10% FBS, 1% antibiotics, and 60 µM 5-fluoro-2’-deoxyuridine (Thermo Fisher Scientific) to inhibit the proliferation of non-neuronal cells.^68,69^ The suspension was seeded onto poly-*L*-lysine- and laminin-coated culture surfaces to promote neuronal attachment.

To evaluate the effects of MSC-CM on neurite extension, 2000 TG neurons were seeded in each chamber of an µ-slide 8 Well (ibidi, Munich, Germany). After 3 days of culture, the medium was completely replaced with either 2D MSC-CM or MSC spheroid-CM; unconditioned medium served as a control. To mimic the oxidative stress observed in dry eye disease, 200 µM hydrogen peroxide (H_2_O_2_) was added to each treatment group.^70–72^ Following 48 h of incubation, the cells were fixed in 4% paraformaldehyde and immunostained for βIII-tubulin (Tuj1; GeneTex, Hsinchu, Taiwan). Fluorescence microscopy was used to image stained neurons, and neurite length was quantified using the NeuronJ plugin in ImageJ software.^58,60^

### Evaluation of the effects of MSC-CM on LPS-activated macrophages

RAW264.7 murine macrophages were maintained in DMEM supplemented with 10% FBS. For activation, cells were seeded at a density of 3 × 10^5^ cells per well in 6-well plates. Once adherent, the macrophages were stimulated with 100 ng/mL lipopolysaccharide (LPS; Sigma-Aldrich) for 6 h to induce a proinflammatory phenotype.^59^ The medium was subsequently removed and replaced with CM derived from either conventional 2D MSC cultures or MSC spheroids. Cells cultured with unconditioned medium served as controls.

The cells were then incubated for an additional 24 h to assess the immunomodulatory effects of the MSC-derived secretome. For this purpose, total RNA was extracted from macrophages for qPCR analysis of proinflammatory gene expression using the primers listed in Table S1. In parallel, culture supernatants were collected, and enzyme-linked immunosorbent assays (ELISAs) were used to measure cytokine levels (R&D Systems, Minneapolis, MN, USA).

### Induction of dry eye and the topical application of the MSC-derived secretome in mice

A dry eye model was established in 8-week-old male C57BL/6 mice through the topical administration of 5 μL of 0.05% BAK to the right eye twice daily for seven consecutive days.^73^ This chemical insult reliably induces corneal epithelial disruption, inflammation, and nerve damage, mimicking key pathological features of dry eye disease.^74,75^

Following injury induction, the mice were randomly assigned to three groups using a list randomizer and received one of the following treatments: (1) unconditioned culture medium (control), (2) CM collected from 2D-cultured MSCs, or (3) CM collected from MSC spheroids. Treatments were administered topically to the ocular surface three times per day—at 9:00 AM, 1:00 PM, and 5:00 PM—for an additional 7 days.

Corneal epithelial integrity was assessed by sodium fluorescein staining and examined under a stereo microscope. The cornea was divided into four quadrants, and each was scored using a standardized grading system: 0 = no staining; 1 = mild punctate staining; 2 = moderate punctate staining; 3 = diffuse staining without plaque formation; and 4 = fluorescein-positive epithelial plaque. The total fluorescein staining score was calculated by summing the quadrant scores, yielding a maximum possible score of 16.^74^

To evaluate corneal surface smoothness, a ring-shaped light source was projected onto the corneal surface, and the reflected image was captured using a stereo microscope. Corneal regularity was assessed on the basis of the distortion of the reflected ring pattern and was graded on a six-point scale: 0 = no distortion; 1–4 = progressive distortion in one to four quadrants; and 5 = severe distortion with no recognizable ring.^17,76^ This method provides a semiquantitative assessment of epithelial surface quality, reflecting both healing, and tear film stability.

### Histological analysis

To evaluate corneal nerve regeneration and immune cell infiltration, the mice were euthanized via carbon dioxide inhalation, and the eyes were promptly enucleated and rinsed with cold PBS. Whole globes were fixed in 1.3% paraformaldehyde for 1 h at room temperature.^77^ After fixation, the corneas were carefully excised, permeabilized with 1% Triton X-100 for 30 min, blocked with 5% goat serum in PBS for 1 h, and then incubated overnight at 4°C with primary antibodies against Tuj1 or CD45 (a pan-leukocyte marker; Abcam, Cambridge, MA, USA) diluted in blocking solution. The following day, the samples were washed extensively with PBS and incubated with fluorophore-conjugated secondary antibodies for 2 h at room temperature in the dark. After PBS washes, the stained corneas were mounted with RapiClear 1.47 (SunJin Lab; Hsinchu, Taiwan) and imaged using a confocal laser scanning microscope.

For each cornea, three nonoverlapping regions were imaged. *Z*-stacks were acquired throughout the epithelial layer, and maximum-intensity projections were generated separately for the subbasal nerve plexus (SBNP) and superficial nerve terminal (SNT) on the basis of the anatomical position within the epithelium.^75^ The corneal nerve density was quantified using ImageJ software and is expressed as the percentage of the Tuj1^+^ area relative to the total image field.^75^ CD45^+^ cell infiltration was quantified by manually counting cells in projected images, with values reported as cells per mm^2^.^75^

Alternatively, enucleated eyes were fixed in 4% paraformaldehyde for 48 h, embedded in paraffin, sectioned sagittally, stained with hematoxylin and eosin (H&E), and examined under a light microscope.

### Statistical analysis

All quantitative data were analyzed using GraphPad Prism software (version 10.4.2; GraphPad Software Inc., San Diego, CA, USA) and are presented as the mean ± standard deviation (SD). For comparisons between two groups, an unpaired two-tailed Student’s *t*-test was used. For analyses involving three or more groups, one-way analysis of variance (ANOVA) was performed, followed by Tukey’s post hoc test for multiple comparisons. A *p*-value less than 0.05 was considered statistically significant.

## Results

### The spheroid assembly of MSCs upregulates the expression of neurotrophic and immunomodulatory factors

**Figure 1.**
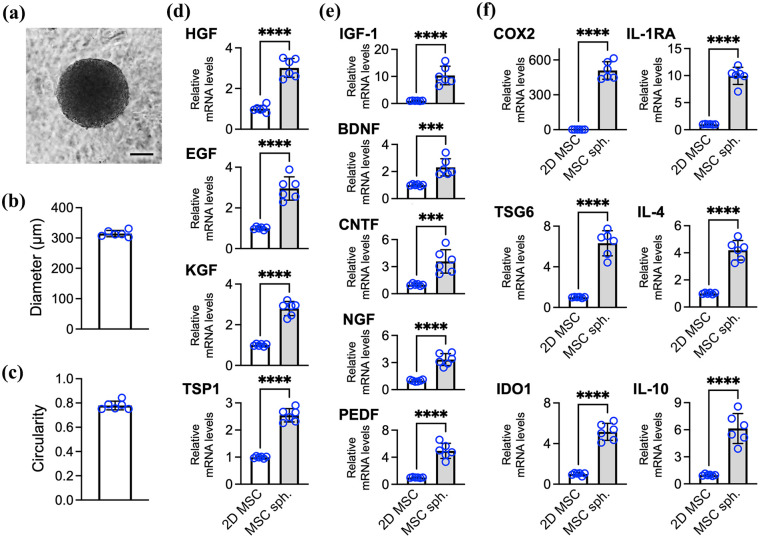
The spheroid assembly of MSCs upregulates the expression of neurotrophic and immunomodulatory factors. (a) Representative morphology of MSC spheroids and quantification of their (b) diameter and (c) circularity. Scale bar, 100 µm. Relative mRNA expression of secreted factors associated with (d) epithelial repair, (e) neurotrophic support, and (f) immunomodulation. The data are presented as the means ± SDs. Statistical significance was determined using Student’s *t* test. ***p < 0.005. ****p < 0.001.

Human MSC spheroids were generated using methylcellulose-coated plates (Figure 1(a)), resulting in highly uniform spheroids with a consistent size (Figure 1(b)) and circular morphology (Figure 1(c)). To evaluate the impact of the 3D configuration on MSC paracrine potential, we compared the gene expression levels of key therapeutic factors between MSC spheroids and conventional 2D cultures. Quantitative PCR analysis revealed significantly elevated transcript levels of factors and enzymes associated with epithelial repair (hepatocyte growth factor (HGF), 3.0-fold, *p* < 0.001; EGF, 2.9-fold, *p* < 0.001; keratinocyte growth factor (KGF), 2.8-fold, *p* < 0.001; thrombospondin-1 (TSP1), 2.5-fold, *p* < 0.001; Figure 1(d)), neurotrophic support (insulin-like growth factor (IGF)-1, 10.4-fold, *p* < 0.001; brain-derived neurotrophic factor (BDNF), 2.3-fold, *p* < 0.005; ciliary neurotrophic factor (CTNF), 3.6-fold, *p* < 0.005; nerve growth factor (NGF), 3.3-fold, *p* < 0.001; pigment epithelium-derived factor (PEDF), 4.9-fold, *p* < 0.001; Figure 1(e)), and immunomodulation (cyclooxygenase-2 (COX2), 509.3-fold, *p* < 0.001; tumor necrosis factor-stimulated gene-6 protein (TSG6), 6.3-fold, *p* < 0.001; indoleamine 2,3-dioxygenase 1 (IDO1), 5.2-fold, *p* < 0.001; IL-1 receptor antagonist (IL-1ra), 9.9-fold, *p* < 0.001; IL-4, 4.2-fold, *p* < 0.001; IL-10, 6.2-fold, *p* < 0.001; Figure 1(f)).

These findings suggest that spheroid formation substantially increases the expression of key therapeutic genes in MSCs, indicating that the resulting secretome is enriched with bioactive factors capable of modulating inflammation and supporting neuroregeneration—two critical targets in the treatment of dry eye disease.

### The spheroid assembly of MSCs enhances the effects of the secretome on corneal epithelial cell proliferation and migration

To investigate the functional impact of spheroid assembly on the therapeutic potential of the MSC-derived secretome, we compared the effects of CM collected from conventional 2D-cultured MSCs and MSC spheroids on corneal epithelial cell proliferation and migration—two key processes involved in epithelial regeneration. To assess the effects under physiological and pathological conditions, human corneal epithelial cells were cultured with either 2D- or spheroid-derived MSC-CM, and proliferation was evaluated under both normal (non-inflammatory) conditions and in an inflammatory microenvironment simulated by IL-1β supplementation.^64,65^

**Figure 2.**
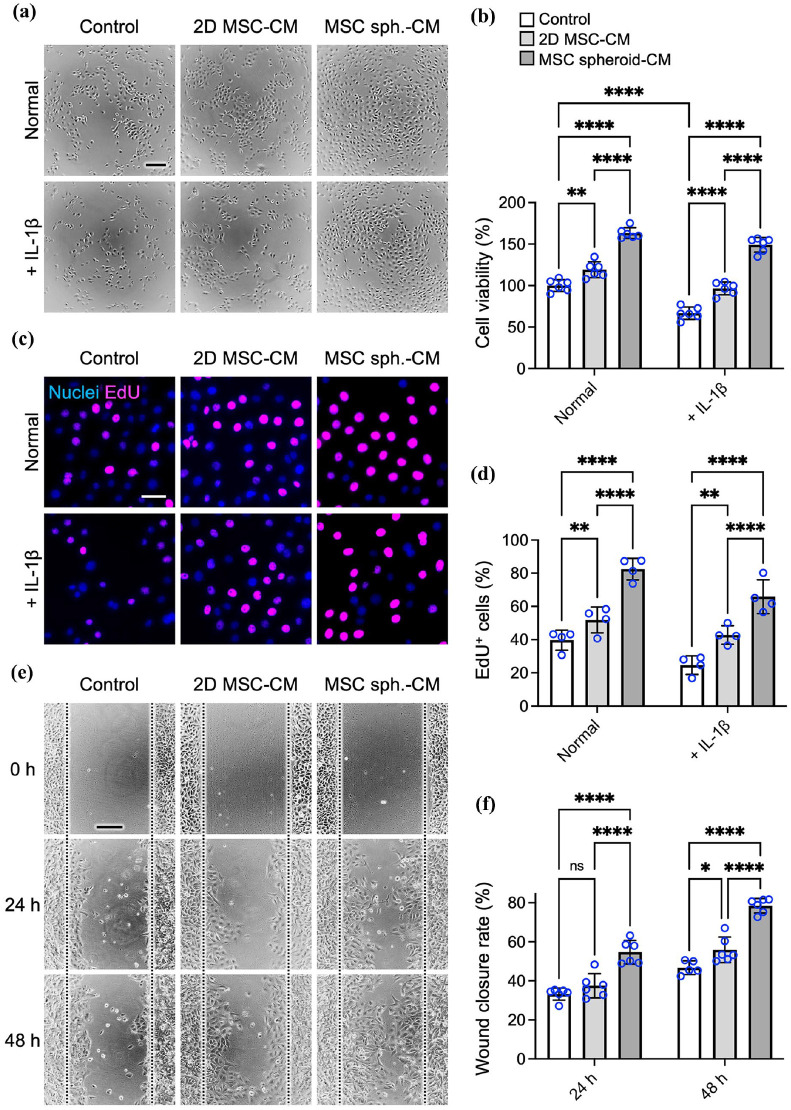
The spheroid assembly of MSCs enhances the effects of the secretome on corneal epithelial cell proliferation and migration. (a) Representative phase-contrast micrographs of corneal epithelial cells cultured with unconditioned medium or CM derived from 2D-cultured MSCs or MSC spheroids under normal conditions and under inflammatory stress induced by IL-1β and (b) the corresponding quantification of corneal epithelial cell proliferation (CCK-8 assays) expressed as a percentage of the control. Scale bar, 200 µm. (c) Representative fluorescence images showing EdU-labeled proliferating cells and (d) quantification of the percentage of EdU^+^ cells. Scale bar, 50 µm. (e) Representative images of the wound healing scratch assay of corneal epithelial cells and (f) the corresponding quantification of the wound closure rate relative to the initial scratch area. The white dashed lines delineate the initial wound border. Scale bar, 200 µm. The data are presented as the means ± SDs. Statistical significance was determined using one-way ANOVA followed by Tukey’s post hoc test. ns: not significant. *p < 0.05. **p < 0.01. ****p < 0.001.

Under normal conditions, compared to unconditioned control medium, both types of MSC-CM significantly increased corneal epithelial proliferation (Figure 2(a) and (b)); the effect was modest with 2D MSC-CM but markedly amplified with MSC spheroid-CM. Specifically, 2D MSC-CM increased cell proliferation by 19.2% ± 6.6% (*p* < 0.01 vs control), whereas MSC spheroid-CM led to a 63.2% ± 9.6% increase (*p* < 0.001 vs control), representing a 3.3-fold increase over 2D MSC-CM (*p* < 0.001; Figure 2(b)).

The advantage of MSC spheroid-CM was even more pronounced under inflammatory stress. Compared with non-inflammatory conditions, exposure to IL-1β significantly suppressed epithelial proliferation (33.3% ± 7.6% reduction; *p* < 0.001; Figure 2(a) and (b)). While 2D MSC-CM partially reversed this suppression (44.6% ± 11.4% increase *vs*. IL-1β-treated control receiving unconditioned medium; *p* < 0.001), MSC spheroid-CM not only restored proliferation but also increased it beyond baseline levels. In the presence of IL-1β, epithelial cell proliferation in the MSC spheroid-CM group was 123.2% ± 15.6% higher than that of control cells treated with unconditioned medium (*p* < 0.001), representing a 2.8-fold greater effect than that observed with 2D MSC-CM under the same conditions (*p* < 0.001; Figure 2(b)).

These findings were further corroborated by EdU staining, which directly labels proliferating cells. Under non-inflammatory conditions, corneal epithelial cells treated with MSC spheroid-CM presented a significantly greater percentage of EdU^+^ cells (82.5% ± 6.5%) than did those treated with 2D MSC-CM (51.8% ± 7.8%; *p* < 0.001) or unconditioned medium (39.7% ± 6.1%; *p* < 0.001; Figure 2(c) and (d)), indicating increased proliferative activity. This proliferative advantage was maintained under IL-1β-induced inflammatory stress, where MSC spheroid-CM-treated cells demonstrated an EdU^+^ ratio of 65.9% ± 10.2%, significantly exceeding that observed with 2D MSC-CM (42.8% ± 5.6%; *p* < 0.001) and unconditioned medium (24.6% ± 6.3%; *p* < 0.001; Figure 2(c) and (d)). Collectively, these results demonstrate that the pro-proliferative activity of the MSC spheroid-derived secretome remains robust in an inflammatory microenvironment.

We next evaluated the effect of MSC-CM on epithelial cell migration using a scratch wound healing assay. Following the creation of a uniform wound in confluent epithelial monolayers, cultures were treated with either 2D- or spheroid-derived MSC-CM and monitored for 48 h. Representative images revealed accelerated wound closure in MSC spheroid-CM-treated cultures, with a minimal residual gap at 48 h, in contrast to slower healing in the 2D MSC-CM and control groups (Figure 2(e)). Quantitative analysis confirmed these observations: the remaining wound area at 48 h was 21.5% ± 3.7% of the original area in the MSC spheroid-CM group, which was significantly lower than that in the 2D MSC-CM group (44.1% ± 6.5%; *p* < 0.001) and the control group (53.3% ± 3.5%; *p* < 0.001; Figure 2(f)).

Together, these findings demonstrate that the MSC spheroid-derived secretome significantly enhances both corneal epithelial cell proliferation and migration in vitro, outperforming the secretome derived from conventional 2D cultures. These effects suggest that MSC spheroid-CM more effectively promotes corneal re-epithelialization, a key process in ocular surface repair in dry eye disease patients.

### The spheroid assembly of MSCs enhances the neurotrophic and neuroprotective potency of the secretome

Corneal nerve damage and denervation are key pathological features of chronic dry eye and contribute significantly to disease progression.^5^ To investigate the potential of the MSC-derived secretome for supporting corneal nerve regeneration, we employed an in vitro model using primary TG neurons, which represent the principal source of corneal innervation.

**Figure 3.**
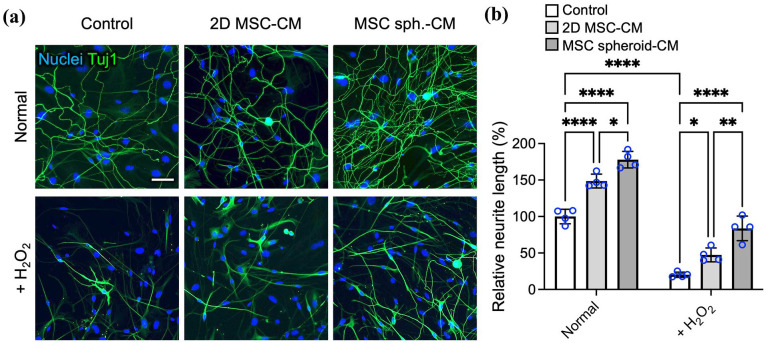
The spheroid assembly of MSCs enhances the neurotrophic and neuroprotective potency of the secretome. (a) Representative immunofluorescence images of Tuj1-stained primary TG neurons cultured with unconditioned medium or CM derived from 2D-cultured MSCs or MSC spheroids under normal conditions and under oxidative stress induced by H_2_O_2_ and (b) the corresponding quantification of average neurite length. Scale bars, 50 µm. The data are presented as the means ± SDs. Statistical significance was determined using one-way ANOVA followed by Tukey’s post hoc test. *p < 0.05. **p < 0.01. ****p < 0.001.

TG neurons were cultured with unconditioned control medium, 2D MSC-CM, or MSC spheroid-CM, and neurite outgrowth was assessed by Tuj1 immunostaining (Figure 3(a)). Compared with the control, both types of MSC-CM significantly promoted neurite extension, with MSC spheroid-CM demonstrating superior efficacy. Quantitative analysis revealed that 2D MSC-CM increased the average neurite length by 48.6% ± 9.5% (*p* < 0.001 vs control), whereas MSC spheroid-CM led to a 77.9% ± 13.4% increase (*p* < 0.001 vs control; *p* < 0.05 vs 2D-CM; Figure 3(b)). These results suggest that assembling MSCs into a spheroid configuration enhances the neurotrophic activity of the derived secretome, which may be critical for promoting corneal nerve regeneration and reinnervation in dry eye disease.

We next evaluated the neuroprotective capacity of the secretome under oxidative stress, a major contributor to inflammation-associated corneal nerve degeneration. TG neurons incubated with unconditioned control medium, 2D MSC-CM, or MSC spheroid-CM were treated with H_2_O_2_ to induce oxidative stress. As shown by Tuj1 staining, H_2_O_2_ exposure caused severe neurite degeneration in control neurons, resulting in a 79.8% ± 3.6% reduction in neurite length relative to that of normal controls without oxidative stress (Figure 3(a) and (b)). Treatment with 2D MSC-CM partially mitigated this effect, reducing neurite loss to 52.6% ± 9.5% (a 2.3-fold improvement over the H_2_O_2_-treated control; *p* < 0.05). In contrast, MSC spheroid-CM provided significantly greater protection, limiting neurite loss to only 16.8% ± 10.3%, corresponding to a 4.2-fold increase in neurite length relative to that of H_2_O_2_-treated controls (*p* < 0.001 vs H_2_O_2_ control; *p* < 0.01 vs 2D MSC-CM; Figure 3(b)).

Together, these findings demonstrate that the MSC-derived secretome promotes neurite outgrowth and confers protection against oxidative stress-induced neuronal damage. Notably, the secretome derived from MSC spheroids exhibits substantially enhanced neurotrophic and neuroprotective potency, supporting its therapeutic potential for facilitating corneal nerve regeneration in dry eye disease.

### The spheroid assembly of MSCs enhances the immunomodulatory capacity of the secretome on macrophages

Chronic dry eye is characterized by persistent inflammation of the ocular surface, which is driven in part by activated macrophages and other immune cells that release proinflammatory cytokines, contributing to tissue damage. MSCs are well known for their immunomodulatory properties and have been applied in various inflammatory disease models, including dry eye. To investigate whether spheroid formation enhances the anti-inflammatory efficacy of the MSC-derived secretome, we evaluated the effects of the MSC-CM on activated macrophages in vitro. Murine RAW264.7 macrophages were stimulated with LPS to induce a proinflammatory phenotype,^9,78–80^ followed by treated with unconditioned control medium, 2D MSC-CM, or MSC spheroid-CM to evaluate the immunomodulatory effects of the secretome.

**Figure 4.**
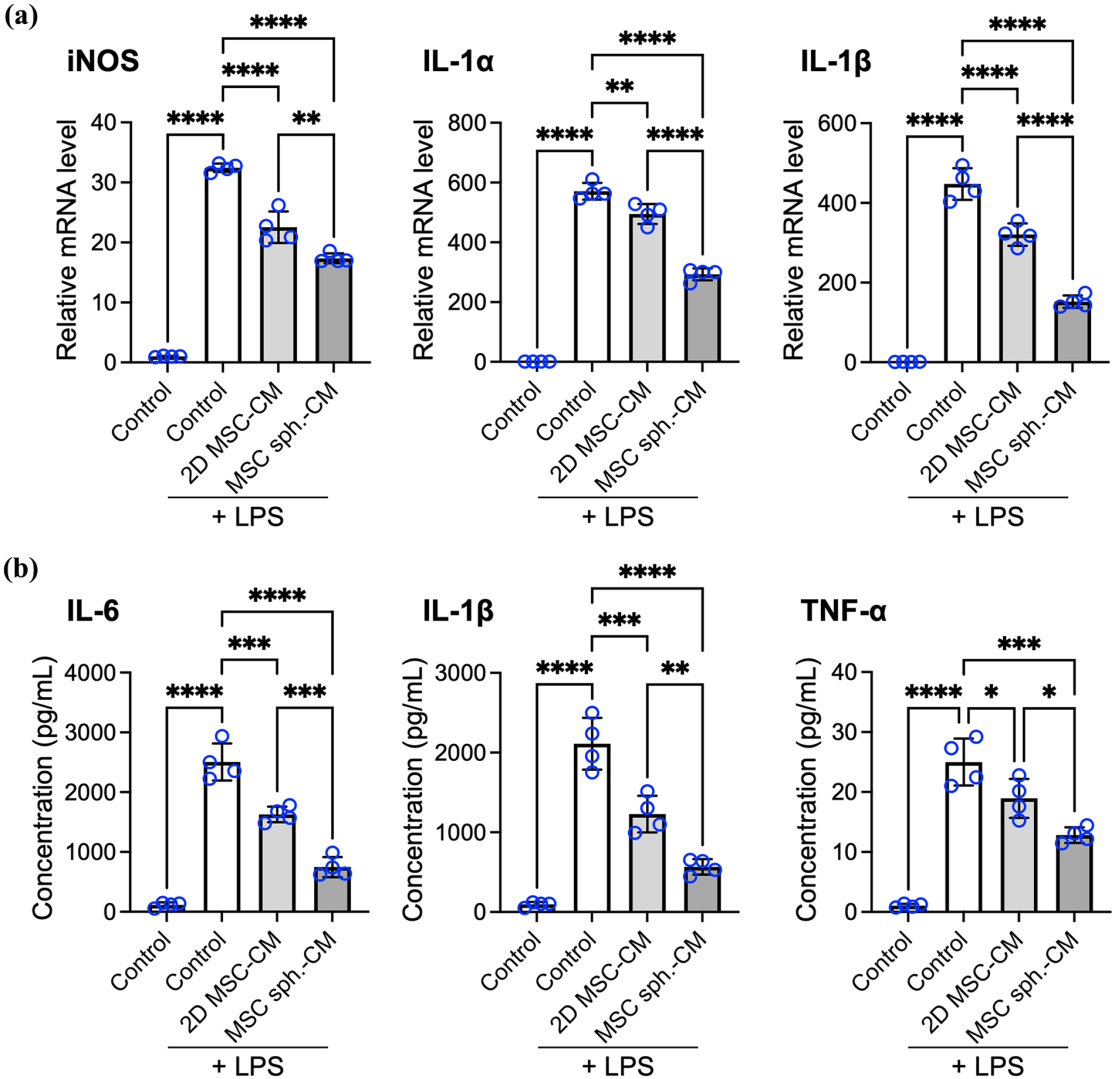
The spheroid assembly of MSCs enhances the immunomodulatory effect of the secretome on macrophages. (a) mRNA expression of proinflammatory markers (iNOS, IL-1α, and IL-1β) in macrophages, as determined by qPCR. (b) Protein levels of inflammatory cytokines released by macrophages, as measured by ELISAs. The data are presented as the means ± SDs. Statistical significance was determined using one-way ANOVA followed by Tukey’s post hoc test. *p < 0.05. **p < 0.01. ***p < 0.005. ****p < 0.001.

We first assessed the mRNA expression of key proinflammatory mediators—inducible nitric oxide synthase (iNOS), IL-1α, and IL-1β—using qPCR (Figure 4(a)). As expected, LPS stimulation markedly upregulated the expression of all three transcripts, confirming successful macrophage activation. Treatment with 2D MSC-CM significantly attenuated this response, reducing iNOS, IL-1α, and IL-1β expression by 30.5% ± 8.2%, 13.3% ± 5.8%, and 28.4% ± 6.3%, respectively (*p* < 0.001, *p* < 0.01, and *p* < 0.001, respectively, compared with the control group treated with LPS and unconditioned medium), indicating the anti-inflammatory potential of the 2D MSC-derived secretome.

Treatment with MSC spheroid-CM led to even greater reductions, decreasing iNOS, IL-1α, and IL-1β transcript levels by 46.5% ± 5.5%, 48.7% ± 5.4%, and 66.1% ± 6.4%, respectively, compared to the control group (*p* < 0.001; Figure 4(a)). Importantly, these reductions were significantly greater than those achieved by 2D MSC-CM (*p* < 0.001; *p* < 0.01 and *p* < 0.001, respectively; Figure 4(a)), highlighting the increased anti-inflammatory potency of the spheroid-derived secretome.

The transcript-level findings were corroborated by ELISAs of secreted cytokines (Figure 4(b)). LPS stimulation led to robust secretion of the IL-6, IL-1β and tumor necrosis factor (TNF)-α proteins, whereas treatment with 2D MSC-CM significantly reduced their levels in the culture supernatant, with reductions of 34.7% ± 5.3%, 41.4% ± 11.5%, and 24.1% ± 4.9%, respectively, compared with those of the LPS-treated control receiving unconditioned medium. Notably, treatment with MSC spheroid-CM resulted in even greater suppression of cytokine release, with reductions of 69.5% ± 10.4%, 72.9% ± 6.1%, and 47.9% ± 10.4% for IL-6, IL-1β, and TNF-α, respectively, relative to the LPS-treated control, and significantly lower levels than those achieved with 2D MSC-CM (*p* < 0.005, *p* < 0.05, and *p* < 0.01, respectively).

Together, the results from the in vitro immunomodulation assays demonstrate that the MSC-derived secretome effectively suppresses macrophage-mediated inflammation and that the secretome derived from MSC spheroids has significantly greater anti-inflammatory potency than the secretome derived from conventional 2D cultures does. These findings suggest that MSC spheroid-CM may more effectively mitigate inflammatory responses on the ocular surface in dry eye disease patients.

Collectively, our in vitro findings show that the MSC spheroid-derived secretome exerts multifaceted therapeutic effects by promoting corneal epithelial proliferation and migration, supporting neurite outgrowth, protecting neurons from oxidative damage, and attenuating macrophage-driven inflammation. These results support the potential of the MSC spheroid-derived secretome as a cell-free regenerative agent capable of alleviating tissue damage and fostering a proregenerative microenvironment in dry eye-affected corneal tissue.

### The spheroid assembly of MSCs enhances secretome-mediated corneal epithelial healing and surface restoration in a BAK-induced dry eye model

**Figure 5.**
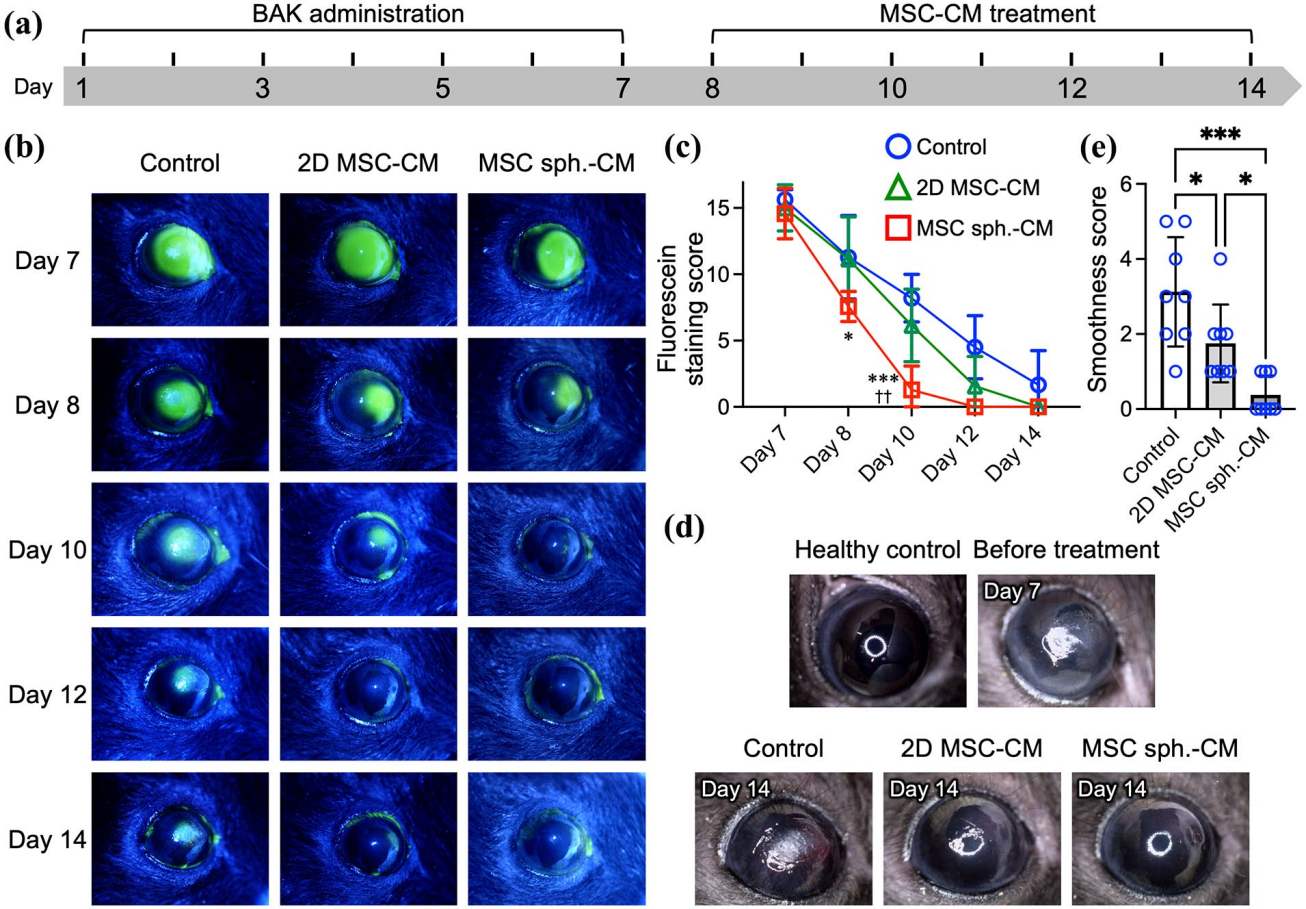
The spheroid assembly of MSCs enhances secretome-mediated corneal epithelial healing and surface restoration in a BAK-induced dry eye model. (a) Experimental timeline of the BAK-induced dry eye model and treatment schedule. (b) Representative images of corneal fluorescein staining from BAK-induced dry eye model mice subjected to different treatments and the corresponding (c) corneal epithelial damage scores. *p < 0.05; ***p < 0.005 vs control; ^††^*p* < 0.01 vs CM-2D. (d) Corneal surface smoothness assessment by ring reflection imaging and (e) the corresponding quantification of corneal smoothness scores for each group on day 14. *p < 0.05; ***p < 0.005. The data are presented as the means ± SDs. Statistical significance was determined using one-way ANOVA followed by Tukey’s post hoc test.

Having established the enhanced regenerative potential of the MSC spheroid-derived secretome in vitro, we next evaluated its therapeutic efficacy in vivo using a BAK-induced dry eye mouse model. The topical application of BAK to the ocular surface for 7 days induced epithelial barrier disruption, surface inflammation, and nerve damage, which are characteristic of dry eye disease (Figure 5(a)). Following injury induction, the mice received three daily topical applications of the MSC secretome for 7 days: one group was treated with MSC spheroid-CM eye drops, another group was treated with 2D MSC-CM eye drops, and a control group was treated with unconditioned medium eye drops (Figure 5(a)). Corneal epithelial healing and surface integrity were assessed across the treatment groups.

Fluorescein staining was conducted at multiple time points to evaluate corneal epithelial defects (Figure 5(b)). On day 7—immediately following BAK exposure and prior to treatment initiation—the mice in all groups exhibited comparable, widespread fluorescein uptake, indicating substantial epithelial disruption. By day 10 (3 days after treatment initiation), eyes treated with control medium continued to display pronounced staining. In contrast, eyes treated with 2D MSC-CM showed moderate improvement, whereas those treated with MSC spheroid-CM exhibited only minimal punctate staining, indicative of marked re-epithelialization.

Corneal fluorescein staining scores corroborated the aforementioned observations (Figure 5(c)). On day 10, for control animals, the staining score was high (8.2 ± 0.3), with 2D MSC-CM-treated mice presenting a modest, nonsignificant reduction (6.1 ± 0.2; *p* > 0.05 vs control); in contrast, mice treated with MSC spheroid-CM presented a significantly lower score of 1.3 ± 0.1 (*p* < 0.005 vs control; *p* < 0.01 vs 2D MSC-CM), indicating nearly complete epithelial restoration. By day 14, all the groups exhibited comparable levels of epithelial healing (*p* > 0.05), which is consistent with the spontaneous recovery typically observed following the cessation of BAK administration, as reported in the literature.^81^ Despite this convergence at the final time point, our findings clearly demonstrate that the MSC spheroid-derived secretome markedly accelerated corneal epithelial wound healing in vivo, outperforming the secretome derived from conventional 2D MSC cultures.

To further evaluate ocular surface integrity following epithelial healing, we assessed corneal smoothness by analyzing the reflection pattern produced by a ring-shaped light source projected onto the corneal surface. In healthy eyes, the reflected image appears as a sharp and continuous ring, indicative of a smooth and intact epithelial surface, whereas epithelial irregularities associated with dry eye produce fragmented and distorted reflections. Prior to treatment (day 7), all BAK-exposed eyes presented highly disrupted ring patterns, whereas untreated healthy eyes presented smooth, uninterrupted reflections (Figure 5(d)). By day 14 (7 days after treatment initiation), eyes treated with control medium continued to show pronounced irregularities. Treatment with 2D MSC-CM led to moderate improvement, with partially restored but still discontinuous ring patterns (Figure 5(d)). In contrast, eyes treated with MSC spheroid-CM exhibited near-normal ring reflections with minimal distortion, indicating substantial restoration of corneal surface smoothness (Figure 5(d)).

Corneal surface smoothness was quantified on day 14 using a surface grading index (Figure 5(e)), in which lower scores indicate smoother surfaces. For MSC spheroid-CM-treated eyes, the average score was 0.38 ± 0.15, which was significantly lower than that of the 2D MSC-CM group (1.75 ± 0.25, *p* < 0.05) and the control group (3.13 ± 0.85, *p* < 0.005). The smoothness score for MSC spheroid-CM-treated corneas closely approximated that of normal, uninjured eyes, underscoring the superior therapeutic efficacy of the MSC spheroid-derived secretome and highlighting its potential as a cell-free therapeutic strategy for ocular surface reconstruction in dry eye disease.

### The spheroid assembly of MSCs enhances the therapeutic efficacy of the secretome in promoting corneal reinnervation and suppressing inflammatory cell infiltration in a dry eye disease model

**Figure 6.**
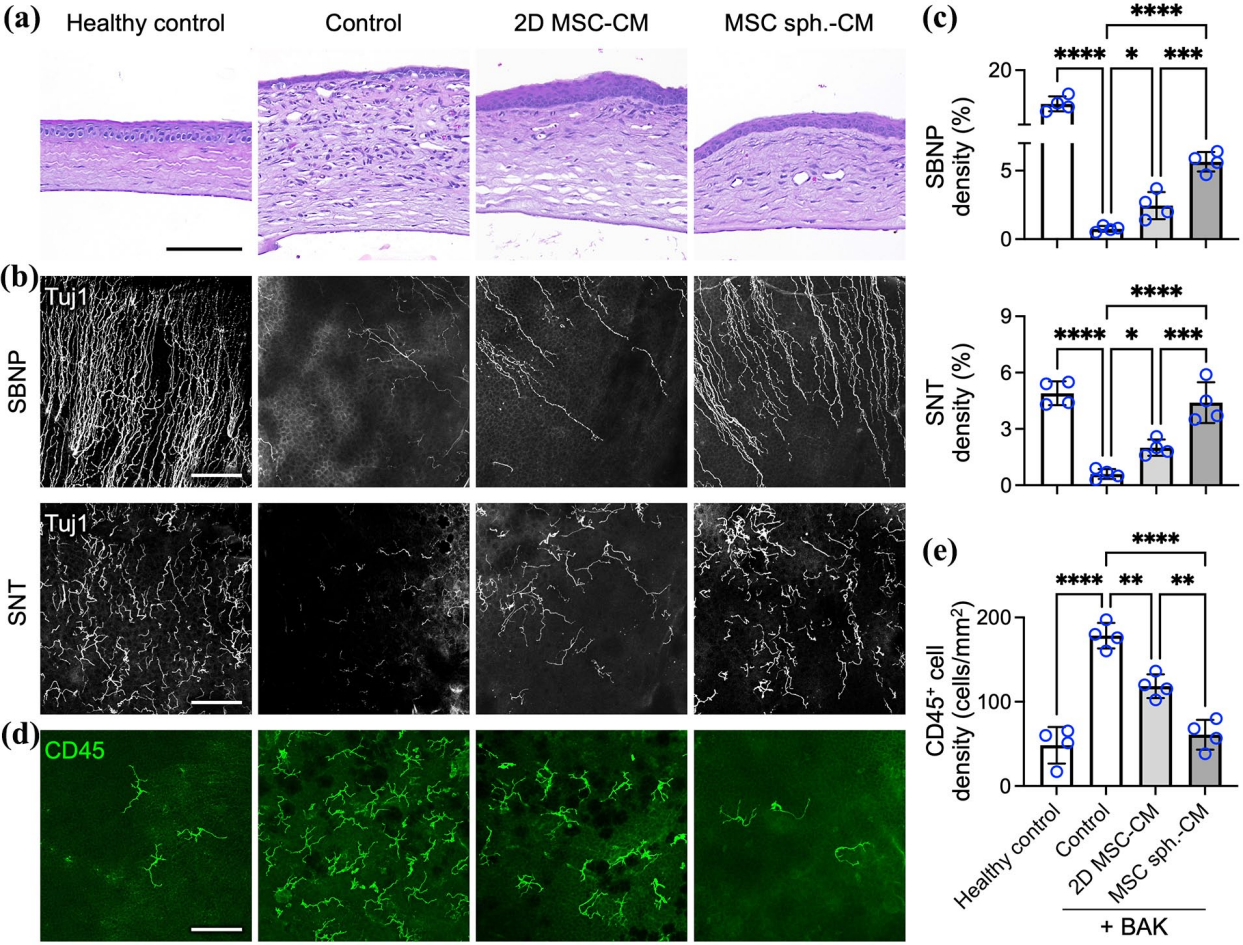
The spheroid assembly of MSCs enhances the therapeutic efficacy of the secretome in promoting corneal reinnervation and suppressing inflammatory cell infiltration in a dry eye disease model. (a) Representative H&E-stained images of corneal sections collected on day 14. (b) Confocal microscopy images of corneal whole mounts collected on day 14 and stained for Tuj1 to visualize corneal nerves, including the SBNP and SNT, and (c) the corresponding quantification of corneal nerve fiber density, expressed relative to the area of the cornea. (d) Representative immunofluorescence images of corneal whole mounts collected on day 14 and stained for the pan-leukocyte marker CD45 to show inflammatory cell infiltration in the cornea and (e) the corresponding quantification of infiltrating CD45^+^ cells in the cornea for each group. Scale bars, (a) 500 µm; (b and d) 100 µm. The data are presented as the means ± SDs. Statistical significance was determined using one-way ANOVA followed by Tukey’s post hoc test. *p < 0.05. **p < 0.01. ***p < 0.005. ****p < 0.001.

To further characterize the therapeutic effects of the MSC secretome, we assessed corneal pathology using histological analysis. H&E staining revealed pronounced stromal edema and mixed inflammatory cell infiltration in animals subjected to 7 days of BAK exposure followed by treatment with unconditioned medium (Figure 6(a)). In contrast, eyes treated with either 2D MSC-CM or MSC spheroid-CM exhibited markedly reduced edema and inflammation, with the spheroid-CM group demonstrating the most substantial histological improvement (Figure 6(a)).

We next used immunofluorescence staining to evaluate corneal reinnervation and inflammatory cell infiltration more closely. Mouse corneas were stained for the neuronal marker Tuj1 and imaged by confocal microscopy to visualize corneal nerve architecture. In normal, uninjured corneas, we observed a dense SBNP with extensive branching, in addition to numerous fine SNT near the epithelial surface (Figure 6(b)). At the end of the BAK induction phase (day 7), prior to treatment initiation, corneal whole mounts from all groups revealed a nearly complete loss of nerve fibers (Figure S1), confirming that BAK exposure reliably induced severe corneal denervation, which is consistent with its well-documented neurotoxic effects in dry eye models.^81^

Following 7 days of treatment (day 14), distinct differences in corneal nerve regeneration were observed among the groups. BAK-treated corneas receiving unconditioned control medium exhibited persistent denervation, with only sparse or absent nerve fibers. Corneas treated with 2D MSC-CM showed partial nerve fiber recovery, whereas those treated with MSC spheroid-CM displayed a markedly denser and more organized nerve network, indicative of enhanced reinnervation (Figure 6(b)).

Quantitative analysis of nerve density, defined as the percentage of the image area occupied by corneal nerves, revealed that BAK exposure followed by control medium treatment resulted in a 94.2% reduction in SBNP density (0.8% ± 0.2%) and an 87.8% reduction in SNT density (0.6% ± 0.3%) compared with the SBNP and SNT densities in healthy controls (13.9% ± 1.4% for SBNP and 4.9% ± 0.6% for SNT; *p* < 0.001 for both; Figure 6(c)). Compared with the control, treatment with 2D MSC-CM promoted significant nerve regeneration, increasing the SBNP and SNT densities by 3.4-fold (2.5% ± 1.0%, *p* < 0.05) and 3.3-fold (2.0% ± 0.4%, *p* < 0.05), respectively. In contrast, MSC spheroid-CM induced markedly greater reinnervation, increasing SBNP and SNT densities by 7.7-fold (5.7% ± 0.7%) and 7.3-fold (4.4% ± 1.1%), respectively (*p* < 0.001 vs control for both; Figure 6(c)); both values were also significantly greater than those observed with 2D MSC-CM (*p* < 0.005 for both), confirming the superior neurotrophic efficacy of the MSC spheroid-derived secretome. These findings suggest that MSC spheroid-CM may stimulate axonal outgrowth and contribute to the preservation of existing nerve fibers, both of which are crucial for alleviating dry eye–induced corneal denervation and, consequently, for effective dry eye management.

In parallel, ocular surface inflammation was evaluated by staining corneal tissue for CD45, a pan-leukocyte marker that identifies infiltrating immune cells. Normal corneas had few CD45^+^ cells, whereas untreated BAK-injured corneas presented extensive leukocyte infiltration (Figure 6(d)), a finding that was consistent with a pronounced inflammatory response. Compared with the control, treatment with 2D MSC-CM reduced the CD45^+^ cell density, with a further reduction in CD45^+^ cells observed after treatment with MSC spheroid-CM (Figure 6(d)), indicating a substantially attenuated inflammatory response.

The quantification of CD45^+^ cells (Figure 6(e)) revealed that BAK-induced injury markedly increased leukocyte infiltration to 178.4 ± 15.2 cells/mm^2^, which was significantly greater than the baseline value of 48.4 ± 21.7 cells/mm^2^ observed in healthy corneas (*p* < 0.001). Treatment with 2D MSC-CM reduced immune cell infiltration by 33.6%, lowering the CD45^+^ cell density to 118.5 ± 14.1 cells/mm^2^ (*p* < 0.01 vs control). Notably, MSC spheroid-CM induced a significantly greater anti-inflammatory effect, decreasing the CD45^+^ cell density by 65.9% to 60.8 ± 17.7 cells/mm^2^ (*p* < 0.001 vs control; *p* < 0.01 vs 2D MSC-CM), underscoring the enhanced immunomodulatory efficacy of the spheroid-derived secretome in vivo.

In summary, these in vivo findings demonstrate that the MSC spheroid-derived secretome is significantly more effective than the secretome derived from conventional 2D MSC cultures in promoting corneal repair in a dry eye disease model. In addition to accelerating epithelial wound healing and restoring surface smoothness, MSC spheroid-CM markedly enhanced corneal nerve regeneration and attenuated leukocyte infiltration. These coordinated therapeutic effects support the restoration of corneal structural integrity and underscore the potential of the MSC spheroid-derived secretome as a promising cell-free strategy for treating dry eye disease.

## Discussion

The present study aimed to evaluate whether MSC spheroid-derived secretome could provide a more potent, cell-free treatment for dry eye disease. Our results demonstrated that the assembly of MSCs into spheroids substantially enhances the therapeutic efficacy of their secretome, resulting in robust improvements in corneal repair in a preclinical model of dry eye disease. Compared with the secretome derived from conventional 2D MSC cultures, the MSC spheroid-derived secretome more effectively promoted corneal epithelial regeneration, restored surface smoothness, enhanced nerve reinnervation, and reduced leukocyte infiltration. These outcomes are particularly compelling given the multifactorial pathogenesis of dry eye, in which epithelial disruption, chronic inflammation, and neurotrophic dysfunction perpetuate a self-reinforcing cycle. By concurrently addressing these interdependent mechanisms, the MSC spheroid-derived secretome offers a biologically integrative and potentially disease-modifying therapeutic strategy. Clinically, this approach holds promise for achieving more complete and sustained restoration of ocular surface integrity than current symptomatic treatments.

To achieve such multi-target benefits, various MSC priming strategies have been developed to increase the regenerative potency of the secretome.^82,83^ Many of these methods rely on the use of exogenous bioactive molecules, growth factors, or pharmacologic agents.^51,83^ Although effective, these approaches may introduce residual elements that require additional purification steps to ensure safety—particularly for sensitive applications such as ocular surface therapy. In contrast, the spheroid culture method employed in this study represents a simple, non-pharmacological, and scalable alternative that avoids the need for external agents^84^; it can be readily implemented using commercially available and clinically compatible culture platforms, increasing its translational relevance.

The superior efficacy of the spheroid-derived secretome is likely attributed to the enhanced paracrine profile conferred by the 3D culture environment. Within spheroids, MSCs undergo extensive cell–cell interactions and are exposed to physiological microenvironments that better mimic native tissue conditions, including mild hypoxia and endogenous extracellular matrix cues.^46–48^ These conditions are known to upregulate a broad array of therapeutic factors, including proregenerative and anti-inflammatory molecules,^52,54,82,85–87^ contributing to improved regenerative outcomes. Specifically, factors that support corneal repair are upregulated in spheroids, including epithelial-supportive growth factors (HGF, EGF, KGF, and TSP1),^64,88,89^ which support epithelial healing; neurotrophic molecules (IGF-1, BDNF, CNTF, NGF, and PEDF),^2,67,90–92^ which support corneal nerve repair; immunomodulatory mediators (TSG6, IL-1ra, IL-4, and IL-10),^37,93–95^ which modulate immune cells; and enzymes involved in the production of anti-inflammatory signals (COX-2 and IDO1).^33,96^

Among these, BDNF, CNTF, HGF, NGF, and PEDF are of particular interest, as they have been previously implicated in promoting corneal nerve regeneration through distinct neurotrophic and anti-apoptotic mechanisms.^2,64,67,90–92^ Given our findings of enhanced nerve regrowth following spheroid-CM treatment, it is plausible that these factors, individually or in combination, contribute meaningfully to the observed neuroregenerative effects. Further studies aimed at identifying the key bioactive constituents will help elucidate the underlying mechanisms and guide the development of more targeted therapeutic strategies. Furthermore, MSC spheroids release a more diverse and potent array of extracellular vesicles, including exosomes enriched with microRNAs and proteins that collectively drive coordinated tissue repair.^19,29,40,41^ This multifactorial secretome enables the simultaneous modulation of epithelial cells, nerve fibers, and immune cells—key cellular contributors to dry eye pathology—highlighting its superiority over conventional single-agent therapies.

Our findings are consistent with and extend the growing body of evidence supporting the use of MSC-based therapies for ocular surface regeneration. Prior studies employing MSC transplantation or the administration of CM and extracellular vesicles have demonstrated therapeutic benefits, including enhanced corneal wound closure, suppression of inflammatory cytokines, and improved tear film stability.^19,35,39,43^ These effects are largely attributed to the paracrine activity of MSCs, reinforcing the central role of secreted bioactive factors in mediating tissue repair. However, most prior studies did not specifically investigate the efficacy of MSC-derived secretome in restoring corneal innervation—an essential yet often underrecognized component of ocular surface health. Corneal nerves play pivotal roles in maintaining epithelial barrier integrity, regulating tear secretion, and modulating neuroimmune interactions.^3,6^ The damage induced by dry eye disease not only compromises ocular surface homeostasis but also contributes to ongoing disease progression.^3,6^

Accumulating evidence, including our previous work^53^ and that of others,^86,97^ has demonstrated that spheroid assembly significantly enhances the neurotrophic potential of MSCs, promoting neurite outgrowth in vitro and supporting axonal regeneration in models of both central and peripheral nervous system injury. Building upon these findings, the present study is, to our knowledge, the first to demonstrate that the MSC spheroid-derived secretome effectively promotes corneal nerve regeneration in the context of dry eye disease. The ocular surface provides a uniquely accessible and localized treatment site, allowing repeated, non-invasive delivery of cell-free therapeutics via topical eye drops.^98^ Within this framework, the enhanced neuroregenerative activity observed in this study is consistent with previous findings in neural tissues and underscores the importance of the MSC configuration in shaping secretome potency.

Moreover, the implications of these findings likely extend beyond dry eye disease alone. Severe corneal injuries, including chemical burns, neurotrophic keratitis, and radiation-induced damage, often involve concurrent epithelial disruption, nerve degeneration, and inflammation.^4,32^ These conditions share key pathological features with dry eye, albeit in more severe forms. Conventional therapies that address only one aspect of the injury often fail to fully restore ocular surface integrity. In contrast, the MSC spheroid-derived secretome, by delivering a concentrated and synergistic repertoire of regenerative factors, may offer a multifaceted therapeutic alternative capable of accelerating epithelial repair, modulating inflammation, promoting reinnervation, and ultimately preserving vision where conventional therapies fall short. While further investigations are needed to validate the efficacy in these severe injury models, the present findings provide compelling proof of concept for broader applications of this cell-free approach in corneal regenerative medicine.

While this study highlights the therapeutic potential of the MSC spheroid-derived secretome for the treatment of dry eye disease, several limitations must be considered before its use in the clinic. First, the BAK-induced dry eye model in mice reproduces only selected aspects of human disease, such as epithelial injury, nerve loss, and inflammation; it does not capture other key features, including autoimmune-driven lacrimal gland dysfunction or evaporative tear deficiency.^99,100^ Additionally, as an acute, chemically induced model, it does not fully capture the chronic and progressive pathophysiology characteristic of clinical dry eye disease. Nevertheless, the favorable outcomes observed in this dry eye model support the therapeutic potential of the MSC spheroid-derived secretome for treating ocular surface disorders. Future studies using alternative models, such as chemical or mechanical injury to the cornea, may provide additional validation and help extend its application to broader regenerative settings.

Second, the relatively short duration of the in vivo experiments restricted our ability to evaluate long-term efficacy and safety, which are critical considerations in managing chronic conditions such as dry eye. Furthermore, the absence of in vivo corneal confocal microscopy represents a methodological shortcoming, as it could provide more comprehensive validation of corneal nerve regeneration. Third, although the study demonstrated morphological nerve regeneration, functional assessments were not performed, leaving it unclear whether structural improvements translate into meaningful recovery of corneal sensitivity or tear reflexes. In addition, while LPS stimulation of macrophages is a commonly used approach to model inflammatory responses, it may not fully capture the complex and multifactorial immune microenvironment associated with dry eye disease.

Moreover, additional work is needed to optimize therapeutic parameters such as spheroid size, incubation time, dosing concentration, and treatment frequency. There is also a need to establish standardized protocols for spheroid fabrication and secretome collection to ensure reproducibility and consistency across studies. Finally, the specific bioactive components responsible for the therapeutic effects observed in the MSC spheroid-derived secretome remain unidentified. Characterizing these active constituents could further clarify the mechanisms underlying our observed effects and support the development of more targeted and scalable regenerative therapies.

## Conclusion

In conclusion, this study provides the first evidence that the secretome derived from MSCs can promote corneal nerve regeneration in a dry eye disease model. Moreover, employing MSC spheroid culture significantly increases the therapeutic efficacy of the secretome, enabling more effective targeting of the key pathological features of dry eye. In our preclinical model, the spheroid-derived secretome markedly accelerated epithelial repair, increased nerve regeneration, and reduced ocular surface inflammation. These coordinated effects underscore the therapeutic advantages of spheroid-derived secretome formulation over conventional monolayer-derived secretome formulations. With further optimization and validation, MSC spheroid-derived secretome therapy holds strong promise as a practical, cell-free strategy for managing dry eye and other ocular surface disorders.

## Supplemental Material

sj-docx-1-tej-10.1177_20417314251363300 – Supplemental material for Spheroid assembly of mesenchymal stem cells enhances secretome-mediated corneal reinnervation and epithelial repair in a mouse model of experimental dry eye Supplemental material, sj-docx-1-tej-10.1177_20417314251363300 for Spheroid assembly of mesenchymal stem cells enhances secretome-mediated corneal reinnervation and epithelial repair in a mouse model of experimental dry eye by Shao-Wen Liu, Meng-Yu Tsai, Yang-Chun Shen, Yi-Jen Hsueh, Han Chiu, Li-Wen Hsu, Hung-Chi Chen and Chieh-Cheng Huang in Journal of Tissue Engineering
