# Supplementary material for: Spheroid assembly of mesenchymal stem cells enhances secretome-mediated corneal reinnervation and epithelial repair in a mouse model of experimental dry eye
Source: J Tissue Eng. 2025 Aug 19;16:20417314251363300. doi: 10.1177/20417314251363300 (PMC12365436; doi:10.1177/20417314251363300)
Supplement: sj-docx-1-tej-10.1177_20417314251363300 – Supplemental material for Spheroid assembly of mesenchymal stem cells enhances secretome-mediated corneal reinnervation and epithelial repair in a mouse model of experimental dry eye [file sj-docx-1-tej-10.1177_20417314251363300.docx]

**
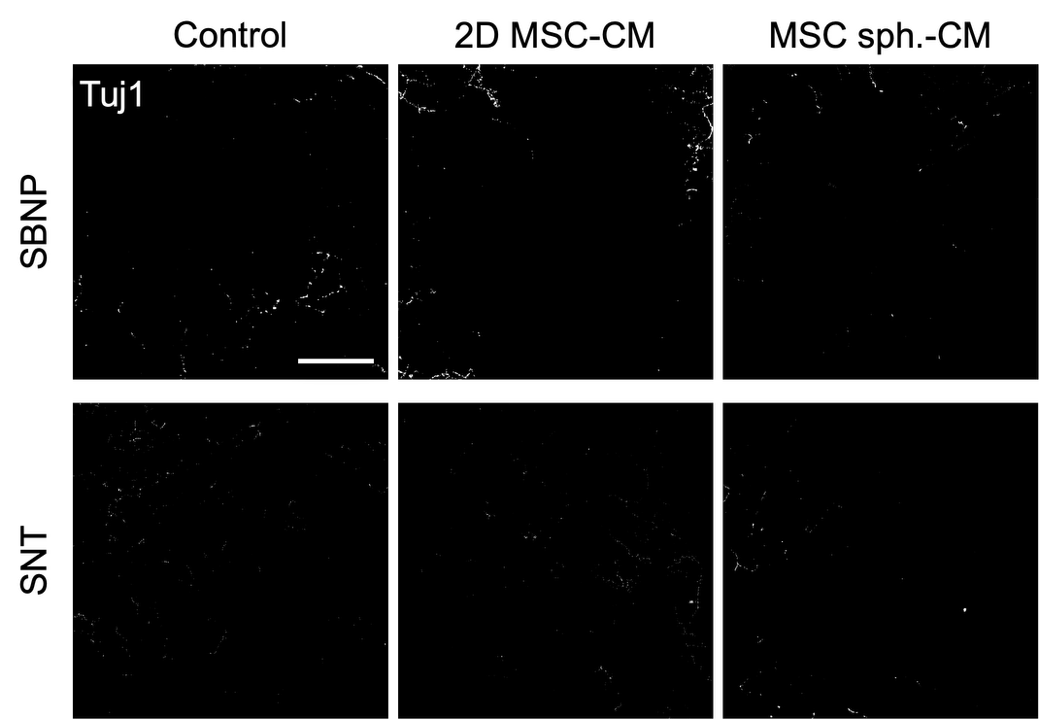
**

**Figure S1.** Confocal microscopy images of corneal whole mounts collected immediately after seven days of BAK exposure and prior to treatment initiation, stained for Tuj1 to visualize corneal nerves, including the subbasal nerve plexus (SBNP) and superficial nerve terminals (SNT). Scale bar, 100 µm.

**Table S1**. Primer sequence used for qPCR.

| **Species** | **Gene** | **Forward** | **Reverse** |
| --- | --- | --- | --- |
| Human | *BDNF* | 5’-GTCAAGTTGGGAGCCTGAAATAGTG-3’ | 5’-AGGATGCTGGTCCAAGTGGTG-3’ |
|  | *CNTF* | 5’-ATGGCTTTCACAGAGCATT-3’ | 5’-AACTGCTACATTTTCTTGTTGTT-3’ |
|  | *EGF* | 5’-CTTGTCATGCTGCTCCTCCTG-3’ | 5’-TGCGACTCCTCACATCTCTGC-3’ |
|  | *FGF7* | 5’-ATCAGGACAGTGGCAGTTGGA | 5’-AACATTTCCCCTCCGTTGTGT-3’ |
|  | *HGF* | 5’-AAGGTGACTCTGAATGAGTC-3’ | 5’-GGCACATCCACGACCAGGAACAATG-3’ |
|  | *IDO1* | 5’-GCCCTTCAAGTGTTTCACCAA-3’ | 5’-GCCTTTCCAGCCAGACAAATAT-3’ |
|  | *IGF1* | 5’-GGTGGATGCTCTTCAGTTCGTG-3’ | 5’-AAATGTACTTCCTTCTGGGTCTTGG-3’ |
|  | *IL1RN* | 5’-AAGATGTGCCTGTCCTGTGTCAA-3’ | 5’-GTTCTCGCTCAGGTCAGTGATGTTA-3’ |
|  | *IL4* | 5’-CCGTAACAGACATCTTTGCTGCC-3’ | 5’-GAGTGTCCTTCTCATGGTGGCT-3’ |
|  | *IL10* | 5’-GACTTTAAGGGTTACCTGGGTTG-3’ | 5’-TCACATGCGCCTTGATGTCTG-3’ |
|  | *NGF* | 5’-AGCGTCCGGACCCAATAACA-3’ | 5’-CCTGCAGGGACATTGCTCTC-3’ |
|  | *PEDF* | 5’-CTGCAGGGACTTGGTGACTT-3’ | 5’-GTCGGACCCTAAGGCTGTTT-3’ |
|  | *PTGS2* | 5’-GAATGGGGTGATGAGCAGTT-3’ | 5’-CAGAAGGGCAGGATACAGC-3’ |
|  | *RPL13A* | 5’-CATAGGAAGCTGGGAGCAAG-3’ | 5’-GCCCTCCAATCAGTCTTCTG-3’ |
|  | *THBS1* | 5’-AGACTCCGCATCGCAAAGG-3’ | 5’-TCACCACGTTGTTGTCAAGGG-3’ |
|  | *TNFAIP6* | 5’-GATGGATGGCTAAGGGCAGAGT-3’ | 5’-TCATTTGGGAAGCCTGGAGATT-3’ |
| Mouse | *Gapdh* | 5’-CTGCCACCCAGAAGACTGTG-3’ | 5’-GGTCCTCAGTGTAGCCCAAG-3’ |
|  | *Il1a* | 5’-TCTCAGATTCACAACTGTTCGTG-3’ | 5’-AGAAAATGAGGTCGGTCTCACTA-3’ |
|  | *Il1b* | 5’-GCCCATCCTCTGTGACTCAT-3’ | 5’-AGGCCACAGGTATTTTGTCG-3’ |
|  | *Nos2* | 5’-GCTTGTCTCTGGGTCCTCTG-3’ | 5’-CTCACTGGGACAGCACAGAA-3’ |
